# Individual Exposure to NO_2_ in Relation to Spatial and Temporal Exposure Indices in Stockholm, Sweden: The INDEX Study

**DOI:** 10.1371/journal.pone.0039536

**Published:** 2012-06-20

**Authors:** Tom Bellander, Janine Wichmann, Tomas Lind

**Affiliations:** 1 Institute of Environmental Medicine, Karolinska Institutet, Stockholm, Sweden; 2 Centre of Occupational and Environmental Medicine, Stockholm County Council, Stockholm, Sweden; 3 School of Health Systems and Public Health, Faculty of Health Sciences, University of Pretoria, Pretoria, South Africa; 4 Section of Environmental Health, Institute of Public Health, University of Copenhagen, Copenhagen, Denmark; Chancellor College, University of Malawi, Malawi

## Abstract

Epidemiology studies of health effects from air pollution, as well as impact assessments, typically rely on ambient monitoring data or modelled residential levels. The relationship between these and personal exposure is not clear. To investigate personal exposure to NO_2_ and its relationship with other exposure metrics and time-activity patterns in a randomly selected sample of healthy working adults (20–59 years) living and working in Stockholm. Personal exposure to NO_2_ was measured with diffusive samplers in sample of 247 individuals. The 7-day average personal exposure was 14.3 µg/m^3^ and 12.5 µg/m^3^ for the study population and the inhabitants of Stockholm County, respectively. The personal exposure was significantly lower than the urban background level (20.3 µg/m^3^). In the univariate analyses the most influential determinants of individual exposure were long-term high-resolution dispersion-modelled levels of NO_2_ outdoors at home and work, and concurrent NO_2_ levels measured at a rural location, difference between those measured at an urban background and rural location and difference between those measured in busy street and at an urban background location, explaining 20, 16, 1, 2 and 4% (R^2^) of the 7-day personal NO_2_ variation, respectively. A regression model including these variables explained 38% of the variation in personal NO_2_ exposure. We found a small improvement by adding time-activity variables to the latter model (R^2^ = 0.44). The results adds credibility primarily to long-term epidemiology studies that utilise long-term indices of NO_2_ exposure at home or work, but also indicates that such studies may still suffer from exposure misclassification and dilution of any true effects. In contrast, urban background levels of NO_2_ are poorly related to individual exposure.

## Introduction

Adverse health effects have been associated with relatively low ambient levels of NO_2_, also below the ambient air quality guideline levels recommended by the World Health Organisation [1]. These health effects include wheezing and exacerbation of asthma, atopy, respiratory infections, reduced lung function, lung cancer, myocardial infarction and death [1]–[5]. The pathogenic mechanisms by which NO_2_ can increase the risk of adverse health outcomes are however not fully understood, but current proposed mechanisms include increased bronchial reactivity and increased susceptibility to bacterial and viral lung infections [1]. It seems however likely that the observed health effects associated with NO_2_ are partly caused by other compounds that are emitted together with NO_2_ or its precursor NO [1].

NO and NO_2_ are by-products of combustion. In Europe and North America, motorised traffic is the main outdoor source of NO_2_ generated in close proximity to people. NO_2_ is often selected as indicator for traffic-related air pollution.

The associations between NO_2_ levels and health effects have been seen both in the temporal domain (time-series and case-crossover studies based on single fixed outdoor monitors) and in the spatial domain (case-referent or cohort studies based on geographically dispersed measurements or geographical modelling). Little is however known about the relationship between these spatiotemporal exposure surrogates and personal exposure [6], [7].

A recent review by Latza et al reported that 93 of 112 epidemiological studies conducted between 2002−2006 linked NO_2_ measured at a single fixed outdoor monitor to health outcomes, whilst others linked personal NO_2_ exposure levels, indoor or outdoor NO_2_ levels at homes [8].

Only few studies have explored the relation between individual exposure and other exposure indices, using large random samples of healthy working adults [9]–[14].

The overall aims of the Individual Exposure to Traffic-related Air Pollution study (INDEX) were to investigate personal exposure to NO_2_ from outdoor environments and to compare personal measurements of healthy working adults to (1) outdoor measurements collected at fixed-site stations (a busy street, urban and rural background) and (2) modelled outdoor estimates at home and work. In addition, the results are useful in estimating the average exposure level in the population as well as to understand how much of the temporal and spatial personal exposure variability can be explained by central monitors and by geographical modelling.

## Methods

In 1999, 1 783 000 people lived in Stockholm County of whom 1 023 000 were 20−59 years old. Of these 175 000 lived in the inner city of Stockholm, and 848 000 outside the inner city, here referred to as ‘outside the city’. In January 1999 a stratified random selection of 4 000 persons aged 20–59 years (born 1940−1979) was conducted. Half of these 4 000 individuals lived in inner city and the rest outside the city.

### Recruitment of Potential Study Participants

**Figure 1:**
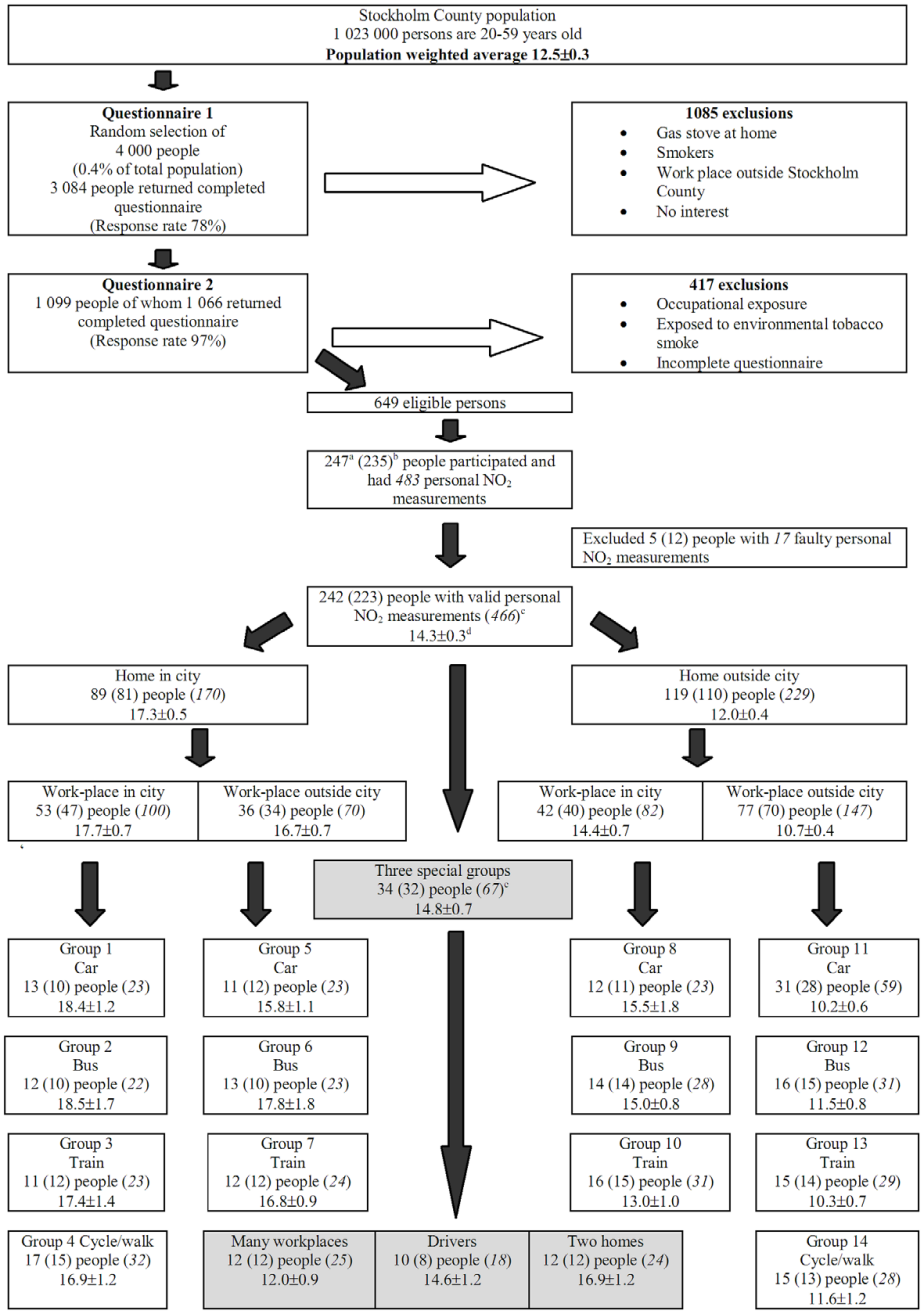
Overview of stratification of 247 study participants from the INDEX study across Stockholm County and estimated NO_2_ levels for different subpopulations. ^a^Number of study participants in the first measurement period. ^b^Number of study participants in the second measurement period (in parenthesis). ^c^Number of valid personal NO_2_ measurements (in parenthesis and italic; during both measurement periods) that was included in the estimation of the weighted population average. ^d^Personal NO_2_ exposure is indicated as mean±standard error of the mean ^e^One person had two personal NO_2_ measurements during the second period.

During February–April 1999 an introduction letter and a short questionnaire were distributed to the 4 000 selected individuals (Figure 1). In total, 3 084 persons (77%) returned their completed questionnaires after two reminders. Of these 1 099 were considered eligible for the study as they reported that they: (1) worked or attended education in Stockholm County, (2) did not have gas stoves at homes, (3) were not smokers and (4) indicated interest to participate in the study. Twenty three percent and 31% of those who were originally approached and lived in and outside Stockholm City fulfilled the four criteria, respectively.

A second questionnaire was sent out to the 1 099 selected participants during March–June 1999. This questionnaire had 15 questions, which collected information on the work address, number of workplaces, working hours, occupational exposure to NO_2_, mode of transport to work, exposure to ETS at home for >6 hours per week and renovations at home. Ninety seven percent of the 1 099 persons returned the completed questionnaires after two reminders. 417 persons were further excluded from the study as they were exposed to NO_2_ at work, lived (or worked) at two addresses (in and outside the city) or were exposed to ETS at home for >6 hours per week.

### Classification of Potential Study Participants

Of the originally selected 4 000 individuals, finally 649 were eligible to be included in the study (Figure 1). These 649 persons were classified into five population strata: work in and living in/outside the city and a special group. Four of the five main strata were then further divided into sub-groups according to the mode of transport to work: travelled to work on foot, by bicycle, bus, car or commuter train. The special group had three sub-groups: taxi drivers and other professional vehicle drivers; people who had many workplaces (4−10 per week) or who had a permanent home in the city, but also stayed occasionally outside the city.

### Selection of Study Participants

The strata were seen as representative of the total eligible population, and in each stratum 12−30 participants were randomly selected to participate in the study, which resulted in a sample size of 247 people. The sampling rate varied from 22 to 80% of the eligible persons per stratum. Personal NO_2_ samplers were mailed to all 247 selected participants during two measurement periods.

Of the selected participants, 28 could not or did not want to participate anymore on the day of their first measurement. They returned the NO_2_ passive samplers and were replaced by people from the same stratum. Those who could not participate gave the following reasons: changes in home, work or family responsibilities since completion of the second questionnaire, moved away from Stockholm County, started to study or parental leave. Of the final study population of 247 persons, 236 had their first measurement during April−September 1999 and 11 had theirs during January−April 2000. During the second measurement period, 235 of the 247 persons participated. Thirty-two of the 235 people had their second measurement during October-December 1999 and the rest theirs during January−April 2000.

### Diary

Instructions were included on how to start and stop the NO_2_ personal measurements, report the start and stop time, how to maintain measurements over 7 days, how to complete the time-activity diaries and how to return the NO_2_ samplers. Participants received a booklet with eight 1-day time schedules, with every day divided in 15-min intervals. For each interval, participants had to mark different activities/locations. The booklet also contained eight 1-day questionnaires regarding specific activities that could influence NO_2_ levels, e.g. cutting grass, fire burning and smoking, and a 1-week time schedule with questions on the week’s work, work place, home conditions and ventilation.

### Field Measurement and Laboratory Analysis of NO_2_

NO_2_ was measured over 7 days using the Swedish Environmental Research Institute (IVL) diffusive samplers [15], [16]. Each personal measurement was started on a Monday morning and stopped the next week Monday at the same time as the start time. A measurement was started by taking the sampler out of a small tube and clipping it onto the outer layer of clothes, as close as possible to the breathing zone. In the evenings the sampler was placed on the bed-side table in the sleeping room.

The samplers were mailed to IVL for analysis by flow injection analysis. All the laboratory measurements for the 1999 field measurements were conducted during May−December 1999, whilst those for 2 000 were conducted during January−May 2000. In total, there were 17 unsuccessful NO_2_ personal measurements: Sampler lost as sampler clip failed, sampler destroyed or lost in mail, or faulty sampling or instructions not followed.

Twenty percent of the personal measurements were performed either in duplicate or complemented by blanks, distributed over the entire study period. Additional information was sent out to participants who performed duplicate or blank measurements.

The observed average field blank was −0.08 µg/m^3^ (*n* = 50, range −0.5−1.6 µg/m^3^, S.D. 0.64 µg/m^3^), after exclusion of one very high field blank (15.1 µg/m^3^). No correction for field blanks was performed. The limit of quantification was 2.0 µg/m^3^, calculated as three times the standard deviation of the field blanks. The lowest recorded NO_2_ personal measurement was 2.4 µg/m^3^.

The average of the duplicate measurements was 19.4 µg/m^3^ (*n* = 50, range 5−49 µg/m^3^). The absolute difference between duplicate measurements was up to 3.6 µg/m^3^. The largest relative difference was 28% (average 7.3%). The estimated coefficient of variation was 5.2%. The variance appeared constant over the range of the duplicate measurements.

### Outdoor Measurements and Dispersion Model Estimates

Urban background (rooftop), street and rural levels were monitored with chemiluminescence monitors by the urban air quality monitoring network of the Stockholm County Council (SLB) [17]. The SLB measurement sites were: Rosenlundsgatan (urban background) and Hornsgatan (busy street) on Södermalm and Aspvreten (rural background). A 7-day average of the urban, street and rural NO_2_ level was calculated for each participant, starting and ending with the hour closest to the start and stop time of the personal measurement.

The *annual* outdoor estimates at each participant’s home and work were estimated with dispersion models, using emission databases and average meteorological parameters [18].

### Statistical Analysis

The data were analysed in two ways: (1) estimating the population NO_2_ exposure levels, and (2) estimating the effect of different determinants on individual exposure.

In the first part the population exposure levels were estimated for each of the 17 predefined population strata (Figure 1). The estimated 7-day population average exposure to NO_2_ among people living in Stockholm County was calculated with respect to the proportion of people in Stockholm belonging to the subgroups and with respect to the repeated measurements on selected participants. This was done by defining subgroups as strata and subjects as clusters. We excluded 17 lost/faulty personal NO_2_ measurements and could use 466 observations. Calculations were done with the *–svy : mean-* command in Stata version 10.

Refer to Text S1 for the tests applied to determine whether personal NO_2_ levels differed significantly (a) between the study participants who lived in the city compared to those who lived outside the city, (b) between those who work in the city compared to those who worked outside the city, after stratifying by home location and (c) across the transport groups, after stratifying by home and work location (Groups 1 to 14, Figure 1).

In the second part we excluded all study participants from the three special exposure groups (drivers and those with multiple workplaces or homes), as no well defined level of outdoor exposure to NO_2_ at work or home could be estimated (Figure S1). We also excluded 61 study participants who had missing data for the independent variables. We did not exclude the 17 study participants who had missing data for the rural background NO_2_ levels, as we observed similar results in the multiple regression models with and without these 17 study participants. Our multiple regression analyses are thus based on 338 valid personal NO_2_ measurements from 175 people. Of the 175 people, 163 had two repeated personal NO_2_ measurements, but not necessarily one measurement in each year (1999 and 2000). Of the 163 people who had the first measurement in 1999, 140 had the repeated measurement in 2000.

Wilcoxon sign-ranked tests were done to test whether personal NO_2_ levels differed significantly from the outdoor urban, street and rural NO_2_ levels and the outdoor NO_2_ estimates at home and work. Spearman rank correlation analyses were performed to determine the correlation coefficients between the outdoor street, urban and rural NO_2_ levels, and the outdoor NO_2_ estimates at home and work.

In the second part we also analysed the relationship between the 7-day average personal NO_2_ levels and independent variables in univariate and multiple regression models. The independent variables included temporal and spatial indices and time-activity patterns. Temporal variables were 7-day urban, street and rural background NO_2_ measurements at fixed central monitoring sites. Spatial variables were dispersion model estimates of *annual* residential and work outdoor NO_2_ levels, and geographical location of home and work: in or outside the city. A large number of variables were extracted from the questionnaires. Included in this presentation are only those that were significantly associated with personal NO_2_ exposure in the univariate models at a 95% confidence level: number of days at work (days/week), total time in transport and at a garage (h/week), time in smoky rooms or rooms with open fire (h/week), time in room with gas appliance (h/week) and sleeping room window facing a large street (yes/no). Refer to Text S2 regarding extreme observations of time spent in places with gas appliances and traffic during the 7-day measurement period. Random effect regression analyses, using the *–xtreg -* command in Stata version 10, were applied to control for within individual correlation due to repeated measurements.

### Ethical Approval

Ethical approval was granted by the KI Research Committee (Document number 98–369). Research was conducted in accordance with principles of the Declaration of Helsinki. Informed consent was obtained from the study participants.

## Results

Figure 1 summarises the observed levels of personal exposure to NO_2_ for people who work and live in different areas in Stockholm and who commute to work by car, commuter train, bus, bicycle or on foot. The 7-day average personal exposure was 14.3 µg/m^3^ for the study population and the estimated 7-day population-weighted average for Stockholm County was 12.5 µg/m^3^, i.e. only about 60% of the observed urban background of 20.3 µg/m^3^ (Table 1).

**Table 1:** Descriptive statistics on individual exposure to NO_2_, temporal and spatial indices and time-activity patterns in Stockholm, Sweden.

Variable	n	Mean±S.D.	Range
7-day average personal NO_2_ exposure (µg/m^3^)	338	14.6±6.3	2.4−40.6
7-day average urban NO_2_ (µg/m^3^)	338	20.3±3.9	9.8−27.7
7-day average street NO_2_ (µg/m^3^)	338	45.5±6.8	35.5−62.9
7-day average rural NO_2_ (µg/m^3^)	321	3.5±1.7	0.5−15.8
Difference between 7-day street and urban NO_2_ (µg/m^3^)	338	25.1±6.5	15.8−40.2
Difference between 7-day urban and rural NO_2_ (µg/m^3^)	338	16.8±4.0	8.2−27.1
Estimated annual average NO_2_ at home (µg/m^3^)	338	14.2±7.7	3.5−34.9
Estimated annual average NO_2_ at work (µg/m^3^)	338	16.7±7.8	3.0−36.2
Number of days at work (days/week)	338	4.5±1.2	0.5−7.0
Time in transport and at a garage (hours/week)	338	10.8±5.0	1.0−32.5
Time in smoky rooms or rooms with open fire (hours/week)	338	4.4±5.9	0−36.3
Time in room with gas appliance (hours/week)	338	0.1±1.3	0−24
Sleeping room window facing a large street	338	5.9%	–
Home located in city	338	42.9%	–
Work located in city	338	44.7%	–

Study participants who lived in the inner city had a personal exposure level (17.3 µg/m^3^) that approached the urban background level (20.3 µg/m^3^, Table 1). As expected, people who lived outside the city had significantly lower levels (12.0 µg/m^3^; *p* = <0.00001; Figure 1). For those who lived outside the city, there was a statistically significant difference according to whether their workplace was in the inner city (14.4 µg/m^3^) or not (10.7 µg/m^3^; *p* = <0.00001). For those who lived in the inner city the location of the workplace did not seem important. Contrary to expectation, the mode of transport to work had no significant influence on personal exposure; although time spent in traffic did. People with two or more homes had on average a significantly higher personal NO_2_ exposure (16.9 µg/m^3^) than the overall average (12.5 µg/m^3^; *p* = 0.006), whilst drivers (14.6 µg/m^3^) (*p* = 0.078) and people with many workplaces (12.0 µg/m^3^) (*p* = 0.493) did not.

Figure S1 summarises the number of participants and personal NO_2_ measurements included in the regression analyses. Table 1 summarises the variables included in the regression analyses. The personal exposure of the 175 study participants (338 measurements) was on average 14.6 µg/m^3^ and varied between 2.4 and 40.6 µg/m^3^ (Table 1). The personal exposure was significantly different from corresponding fixed monitoring levels: about 11 µg/m^3^ higher than the corresponding rural levels and about 31 and 6 µg/m^3^ lower than the street and urban background levels, respectively. The estimated annual outdoor home level was on average very close to the observed 7-day personal level, while the estimate of the annual outdoor level at work was significantly higher by about 2 µg/m^3^. On average, the study participants were 4.5 days at work per week. The average time spent in traffic and filling up a car at a garage was 10.8 h/week, compared to 4.4 h/week spent in smoky rooms and 0.1 h/week spent in rooms with gas appliances (in other places than at home). Less than 6% of the study participants reported having a sleeping room window that faced a busy street.

In the univariate regression of the 7-day average personal NO_2_ levels on possible temporal and spatial factors, the most important variable was estimated annual NO_2_ level at home, which explained 20% (R^2^) of the variation in the observed personal NO_2_ levels (Table 2). Other important determinants were spatial factors such as the home location (17%), annual NO_2_ levels at work (16%) and workplace (9%). Temporal factors such as the 7-day average street and urban NO_2_ levels explained 7% and 3% of the variation in personal NO_2_ levels, respectively. The difference between the 7-day street and urban NO_2_ significantly explained 4% of the variation in the personal NO_2_ levels, whilst difference between the 7-day urban and rural NO_2_ significantly explained 2%. The 7-day average rural background levels did not influence the personal NO_2_ levels. The time-activity variables explained 1–2% of the variation in personal NO_2_ levels. Time spent in traffic was not related to personal NO_2_ levels. When we excluded one extreme value of time spent in traffic (for 32.5 h/week), the variable still did not significantly influence the personal NO_2_ levels (coefficient = 0.09, 95%CI: −0.04−0.23, *p* = 0.182, R^2^ = 0.006). When we excluded one extreme value of time spent in rooms with gas appliances (for 24 h/week), the variable did not significantly influence the personal NO_2_ levels anymore (coefficient = 1.41, 95%CI: −0.40−3.22, *p* = 0.125, R^2^ = 0.002), hence the extreme value influenced personal NO_2_ levels. Having a sleeping room window that faces a busy road explained 3% of the variation in personal NO_2_ levels.

**Table 2:** Univariate models: Relationship between individual exposure to NO_2_, and temporal and spatial indices and time-activity patterns in Stockholm, Sweden.

Model	Coefficient (95% CI)	*p*	R^2^
*Model 1*			0.03
7-day urban NO_2_ (µg/m^3^)	0.27 (0.12−0.41)	<0.0001	
Constant	9.25 (6.24−12.25)	<0.0001	
*Model 2*			0.07
7-day street NO_2_ (µg/m^3^)	0.25 (0.18−0.33)	<0.0001	
Constant	3.30 (−0.22−6.81)	0.066	
*Model 3*			0.04
Difference between 7-day street and urban NO_2_ (µg/m^3^)	0.17 (0.09−0.24)	<0.0001	
Constant	10.56 (8.43−12.69)	<0.0001	
*Model 4*			0.01
7-day rural NO_2_ (µg/m^3^)	0.26 (−0.12−0.65)	0.181	
Constant	13.78 (12.23−15.34)	<0.0001	
*Model 5*			0.02
Difference between 7-day urban and rural NO_2_ (µg/m^3^)	0.24 (0.09−0.40)	0.001	
Constant	10.60 (7.95−13.25)	<0.0001	
*Model 6*			0.20
Estimated annual NO_2_ at home (µg/m^3^)	0.37 (0.28−0.46)	<0.0001	
Constant	9.43 (7.98−10.88)	<0.0001	
*Model 7*			0.16
Estimated annual NO_2_ at work (µg/m^3^)	0.31 (0.22−0.40)	<0.0001	
Constant	9.46 (7.80−11.11)	<0.0001	
*Model 8*			0.01
Number of days at work	0.70 (0.19−1.21)	0.008	
Constant	11.56 (9.16−14.00)	<0.0001	
*Model 9*			
Time in transport and at a garage (hours/week)	0.11 (−0.02−0.24)	0.110	0.01
Constant	13.52 (11.89−15.15)	<0.0001	
*Model 10*			0.02
Time in smoky rooms or rooms with open fire (hours/week)	0.14 (0.03−0.25)	0.009	
Constant	14.07 (13.16−14.99)	<0.0001	
*Model 11*			0.02
Time in room with gas appliance (hours/week)	0.64 (0.20−1.09)	0.004	
Constant	14.60 (13.81−15.40)	<0.0001	
*Model 12*			0.03
Sleeping room window facing a large street	3.21 (0.17−6.24)	0.038	
Constant	14.46 (13.65−15.28)	<0.0001	
*Model 13*			0.17
Home located in city	5.12 (3.68−6.56)	<0.0001	
Constant	12.45 (11.50−13.40)	<0.0001	
*Model 14*			0.09
Work located in city	3.86 (2.36−5.37)	<0.0001	
Constant	12.94 (11.93−13.95)	<0.0001	

Among the variables used in the regression analyses there were weak, but statistically significant correlations between street and urban NO_2_ levels (r^2^ = 0.34), between rural and urban NO_2_ levels (r^2^ = 0.28) and between home and work NO_2_ levels (r^2^ = 0.32). In order to decrease collinearity, the difference between fixed station measurements were used rather than the station values themselves. The difference between the 7-day street and urban NO_2_ levels was not correlated with the difference between the 7-day urban and rural NO_2_ levels (r^2^ = −0.18). Neither were 7-day rural levels correlated with the corresponding difference between urban and rural levels (r^2^ = −0.02). In contrast, the observed urban NO_2_ levels were correlated with the difference in the 7-day street and urban NO_2_ levels (r^2^ = −0.29), and collinearity could not be avoided.

A regression model with the spatial factors annual NO_2_ levels at home and work explained 28% of the variation in personal NO_2_ levels (Model 15 in Table 3), which might be compared with the univariate model with home level alone, which explained 17% (Table 2). As an alternative to the highly resolved spatial modelling data for NO_2_ at home and work, simple classifications of these locations might be considered. Our very simple categories: within or outside the inner city, performed quite well, together explaining 21% of the variation in personal NO_2_ (Model 16 in Table 3). A regression model with NO_2_ levels from rural, urban and street ambient monitoring sites explained marginally more (9%) (Model 17 in Table 3) than with street alone (7%) (Model 2 in Table 2). Combining the spatial and temporal factors in the model explained 31% to 38% of the variation in personal NO_2_ levels (Model 18 in Table 3, Models S4 to S7 in Table S1). This could be somewhat improved by adding questionnaire data. Adding time in traffic improved the R^2^ by 1% (Models S8 and S10 in Table S1), and if excluding the extreme value the variable did not influence personal NO_2_ levels significantly (Models S9 and S11 in Table S1). The location of the sleeping room did not influence personal NO_2_ levels significantly in the multiple regression models (Models S12 and S13 in Table S1), hence the variable was excluded from the multiple regression models. Including only questionnaire variables in the multiple regression model explained 26% of the variation in personal NO_2_ levels (Model 19 in Table 3). Adding the four time-activity variables along with the spatial and temporal factors improved the R^2^ by 6% (Model 20 in Table 3).

**Table 3:** Multiple regression models: Relationship between individual exposure to NO_2_, and temporal and spatial indices and time-activity patterns in Stockholm, Sweden.

Model	Coefficient (95% CI)		*p*	R^2^
*Model 15: Long-term estimates*				0.28
Estimated annual NO_2_ at home (µg/m^3^)	0.30 (0.21−0.39)		<0.0001	
Estimated annual NO_2_ at work (µg/m^3^)	0.23 (0.15−0.32)		<0.0001	
Constant	6.52 (4.80−8.24)		<0.0001	
*Model 16: Location*				0.21
Home located in city	4.46 (3.03−5.89)		<0.0001	
Work located in city	2.77 (1.35−4.20)		<0.0001	
Constant	11.49 (10.4−12.53)		<0.0001	
*Model 17: Concurrent monitoring*				0.09
7-day rural NO_2_ (µg/m^3^)	0.65 (0.28−1.02)		0.001	
Difference between 7-day street and urban NO_2_ (µg/m^3^)	0.24 (0.16−0.33)		<0.0001	
Difference between 7-day urban and rural NO_2_ (µg/m^3^)	0.37 (0.23−0.52)		<0.0001	
Constant	−0.01 (−4.28−4.26)		0.997	
*Model 18: Long-term + monitoring*				0.38
Estimated annual NO_2_ at home (µg/m^3^)	0.31 (0.23−0.40)		<0.0001	
Estimated annual NO_2_ at work (µg/m^3^)	0.23 (0.15−0.31)		<0.0001	
7-day rural NO_2_ (µg/m^3^)	0.57 (0.24−0.90)		0.001	
Difference between 7-day street and urban NO_2_ (µg/m^3^)	0.25 (0.17−0.33)		<0.0001	
Difference between 7-day urban and rural NO_2_ (µg/m^3^)	0.39 (0.25−0.52)		<0.0001	
Constant	−8.47 (−12.66–−4.27)		<0.0001	
*Model 19: Location + questionnaire*				0.26
Home located in city	4.30 (2.88−5.71)	<0.0001		
Work located in city	2.64 (1.23−4.05)	<0.0001		
Time in transport and at a garage (hours/week)	0.09 (−0.03−0.21)	0.146		
Time in room with gas appliance (hours/week)	0.56 (0.15−0.98)	0.007		
Time in smoky rooms or rooms with open fire (hours/week)	0.11 (0.01−0.21)	0.026		
Number of days at work	0.72 (0.25−1.19)	0.003		
Constant	6.92 (4.22−9.62)	<0.0001		
*Model 20: Full model*				0.44
Estimated annual NO_2_ at home (µg/m^3^)	0.30 (0.22−0.38)	<0.0001		
Estimated annual NO_2_ at work (µg/m^3^)	0.23 (0.16−0.31)	<0.0001		
7-day rural NO_2_ (µg/m^3^)	0.53 (0.21−0.85)	0.001		
Difference between 7-day street and urban NO_2_ (µg/m^3^)	0.27 (0.20−0.35)	<0.0001		
Difference between 7-day urban and rural NO_2_ (µg/m^3^)	0.37 (0.24−0.50)	<0.0001		
Time in transport and at a garage (hours/week)	0.13 (0.02−0.24)	0.020		
Time in room with gas appliance (hours/week)	0.45 (0.06−0.84)	0.023		
Time in smoky rooms or rooms with open fire (hours/week)	0.21 (0.12−0.30)	<0.0001		
Number of days at work	0.50 (0.08−0.93)	0.021		
Constant	−13.12 (−17.68–−8.56)	<0.0001		

## Discussion

The main finding of this study is that personal exposure to NO_2_ is related both to spatial factors such as dispersion model estimates of long-term average levels at home and work, and to temporal factors like concurrent ambient monitoring levels. In addition, the estimated average working population exposure level in Stockholm County was considerably lower than the urban background (13 vs 20 µg/m^3^).

A major strength of this study was the large size and well-defined structure of the sample. Great effort was taken to understand from which population stratum the participants originated, and to ensure that important potential determinants were well represented and documented. A related weak point was obviously the self-selection into the study, but the initial response rate was a satisfactory 78%. We excluded smokers and homes with gas stoves, making the results interpretable as individual exposure to ambient NO_2_. Our results should thus not be interpreted as speaking of total exposure to NO_2_. Another strong point was the high quality of the week-long personal NO_2_ measurements, in spite of the self-administration of sampling. The sampling took place over a 13-month period, and most of the participants had one repeated sample.

The spatial factor that best predicted the 7-day average personal exposure was the long-term dispersion modelling estimate of home levels of NO_2_. These dispersion model estimates are based on local emissions and meteorology. This factor alone explained 20% of the inter-individual variability in exposure, in spite of the comparatively short sampling time. This adds credibility to epidemiological studies based on such spatial modelling. In this geographical area we have shown an association between annual home exposure estimates [18] and preschool respiratory disease [3], lung cancer incidence [4] and myocardial infarction mortality [5]. Our findings also indicate that dispersion model estimates of ambient levels at the workplace may be used to further improve exposure classification; however with the addition of such an outdoor estimate for the workplace there might still be substantial misclassification. A very simple spatial indicator of home and workplace location within or outside the city explained differences in individual exposure surprisingly well (R^2^ = 0.21). Mode of transport between home and work did not seem to influence the personal exposure level, which was contrary to expectation.

Because of the short duration of the sampling (7 days), we expected the observed personal levels to be substantially influenced by variation in ambient levels, primarily in urban background. However, none of the observed temporal factors predicted average personal exposure well. There was some correlation to observed concurrent street level of NO_2_, although this level on average was much higher than the individual level. The urban background levels explained less of the variability in individual exposure, which was somewhat unexpected. The reason for this is probably the large spatial variability of NO_2_ [7]. Time in traffic alone did not seem important (i.e. univariate model), which also was contrary to expectation. Although several of the time-activity variables collected in diaries were statistically significant related to personal exposure, the explanatory power was low, and the inclusion of these variables made only a marginal improvement of the multiple regression models based on address-related and fixed monitoring data. We also collected more detailed information on home characteristics like orientation of bedroom window in relation to busy streets and whether the bedroom window usually was kept open. These variables did not seem important in the multiple regression analysis, and other studies have shown that Swedish houses in general are quite permeable to ambient NO_2_ [19]. Thus, in our study very little was gained by the quite laborious collection of time-activity data. One might speculate that street measurements, at least in some cities, being closer to the major source, better capture the combined effect of source strength and ventilation on personal exposure. In most time-series and case-crossover epidemiological studies however, great care is taken to use only urban background monitoring stations, following the reasoning that these are the unavoidable levels for the population. Our observation indicates that for road traffic-related exposures also street-type stations may be considered. This needs to be further explored.

The 7-day average personal exposure of 14.3 µg/m^3^ for our study participants is lower than those reported from six other cities that also focused on randomly selected healthy working adults (Table S2). Part of the difference between studies could be differences in time. The introduction of catalytic converters has brought down NO_2_ levels in many places. Over a period 20 years the annual urban background NO_2_ average in Stockholm decreased by one third, from 30 µg/m^3^ in 1982 to 20 µg/m^3^ in 2002 [17]. However, not all of the studies had been performed before ours (1999/2000). Other potentially important differences obviously include differences in traffic, but also the presence of indoor sources. We excluded smokers and homes with gas stoves, since we were not interested in these contributions to (ambient) pollutant levels in inhaled air. The SAPALDIA study did not control for NO_2_ indoor sources and therefore not surprisingly report a high coefficient of determination between the 7-day personal NO_2_ exposures and 7-day home indoor NO_2_ concentrations [11]. Spengler et al reported that 48% of the variation in 48-h personal exposures was explained by concurrent measured 48-h home outdoor levels [9], which is broadly in line with our findings. Kousa et al reported that Basel, Helsinki and Prague each produced a different regression model [13]. Thus results cannot be extrapolated to other cities. None of the aforementioned six studies included any time-activity pattern variables, rural, urban or street ambient levels in the regression analyses.

From a quantitative view, it is interesting to note that in the full model the coefficients for the ambient or modelled NO_2_ concentrations were between 0.23 and 0.53 (Model 20 in Table 3), i.e. for a unit increase in long-term home or workplace ambient levels or concurrent levels at ambient monitoring sites, the predicted individual exposure increased by about 0.23 to 0.53 units. In this study the variability of these variables is similar, which is why they appear to be equally important for individual exposure.

The usefulness of NO_2_ as an indicator of exposure to the complex mix of exhaust gases and particles has been questioned, since the relation between ambient levels of several of these compounds do not show a linear relation with ambient NO_2_ levels [20]. It would thus be interesting to include personal monitoring of e.g. NO_x_ in future studies of determinants of personal exposure in the population.

In conclusion, short-term personal exposure to NO_2_ was related to dispersion modelled long-term levels at home and work and to a lesser extent to concurrent ambient monitoring levels. This study thus provides a link in the chain between ambient levels and individual exposure, adding credibility primarily to long-term studies based on spatial differences. The results also indicate that such studies may suffer from severe misclassification of exposure and dilution of any true effects. Personal exposure was on average lower than the urban background, which indicates that ecological studies based on large-scale differences, e.g. between cities, overestimate the exposure level for the population, and subsequently also this design underestimates any true effect of a specific individual exposure.

## Supporting Information

Figure S1 **Overview of the number of personal NO_2_ measurements included in the regression analyses.**
(DOC)

Table S1 **Multiple regression models: Relationship between individual exposure to NO_2_, and temporal and spatial indices and time-activity patterns in Stockholm, Sweden.**
(DOC)

Table S2 **Overview of studies of personal exposure to NO_2_ in random samples of healthy working adult populations.**
(DOC)

Text S1 **Tests applied to determine significance of differences in personal NO_2_ levels.**
(DOC)

Text S2 **Extreme observations for time-activity variables.**
(DOC)

## References

[1] World Health Organisation (2006). Air Quality Guideline Global Update 2005: Copenhagen, Denmark.. http://www.euro.who.int/__data/assets/pdf_file/0005/78638/E90038.pdf.

[2] Chen Y, Craig L, Krewski D (2008). Air quality risk assessment and management.. J Toxicol Environ Health Part A.

[3] Nordling E, Berglind N, Melén E, Emenius G, Hallberg J (2008). Traffic-related air pollution and childhood respiratory symptoms, function and allergies.. Epidemiology.

[4] Nyberg F, Gustavsson P, Järup L, Bellander T, Berglind N (2000). Urban air pollution and lung cancer in Stockholm.. Epidemiology.

[5] Rosenlund M, Bellander T, Nordquist T, Alfredsson L (2009). Traffic-generated air pollution and myocardial infarction.. Epidemiology.

[6] Sarnat JA, Wilson WE, Strand M, Brook J, Wyzga R (2007). Panel discussion review: session 1- exposure assessment and related errors in air pollution epidemiologic studies.. J Expo Sci Environ Epidemiol 17.

[7] Monn C (2001). Exposure assessment of air pollutants: a review on spatial heterogeneity and indoor/outdoor/personal exposure to suspended particulate matter, nitrogen dioxide and ozone.. Atmos Environ.

[8] Latza U, Gerdes S, Baur X (2009). Effects of nitrogen dioxide on human health: systematic review of experimental and epidemiological studies conducted between 2002 and 2006.. Int J Hyg Environ Health.

[9] Spengler J, Schwab M, Ryan PB, Colome S, Wilson AL (1994). Personal exposure to nitrogen dioxide in the Los Angeles basin.. J Air Waste Manage Assoc.

[10] Levy JI, Lee K, Spengler JD, Yanagisawa Y (1998). Impact of residential nitrogen dioxide exposure on personal exposure: an international study.. J Air Waste Manag Assoc.

[11] Monn C, Brändli O, Schindler C, Ackerman-Liebrich U, Leuenberger P (1998). Personal exposure to nitrogen dioxide in Switzerland.. Sci Tot Environ.

[12] Lai HK, Kendall M, Ferrier H, Lindup I, Alm S (2004). Personal exposures and microenvironment concentrations of PM_2.5_, VOC, NO_2_ and CO in Oxford, UK.. Atmos Environ.

[13] Kousa A, Monn C, Rotko T, Alm S, Oglesby L (2001). Personal exposures to NO_2_ in the EXPOLIS-study: relation to residential indoor, outdoor and workplace concentrations in Basel, Helsinki and Prague.. Atmos Environ.

[14] Williams R, Jones P, Croghan C, Thornburg J, Rodes C (2011). The influence of human and environmental exposure factors on personal NO_2_ exposures.. J Exp Sci Environ Epidemiol 1–7.

[15] Ferm M, Lindskog A, Svanberg P-A, Boström C-Å (1994). Ny mätteknik för luftföroreningar.. Kemisk Tidskrift.

[16] Ferm M, Svanberg P-A (1998). Cost-efficient techniques for urban and background measurements of SO_2_ and NO_2_.. Atmos Environ.

[17] (2012). SLB-analys vid Miljöförvaltningen i Stockholm. Luften i Stockholm. Årsrapport 2002. Rapport nr 2: 2003.. Accessed on.

[18] Bellander T, Berglind N, Gustavsson P, Jonson T, Nyberg F (2001). Using geographic information systems to assess individual historical exposure to air pollution from traffic and house heating in Stockholm.. Environ Health Perspect 109.

[19] Wichmann J, Lind T, Nilsson MA-M, Bellander T (2010). PM_2.5_, soot and NO_2_ indoor−outdoor relationships at homes, pre-schools and schools in Stockholm, Sweden.. Atmos Environ.

[20] Miller L, Lemke LD, Xu X, Molaroni SM, You H (2010). Intra-urban correlation and spatial variability of air toxics across an international airshed in Detroit, Michigan (USA) and Windsor, Ontario (Canada).. Atmos Environ.

