# Supplementary material for: Individual Exposure to NO2 in Relation to Spatial and Temporal Exposure Indices in Stockholm, Sweden: The INDEX Study
Source: PLoS One. 2012 Jun 20;7(6):e39536. doi: 10.1371/journal.pone.0039536 (PMC3380030; doi:10.1371/journal.pone.0039536)
Supplement: Figure S1 — Overview of the number of personal NO2 measurements included in the regression analyses. (DOC) [file pone.0039536.s001.doc]

| **First measurement period**  April−September 1999 and January−April 2000 | | |  | **Second measurement period**  October−December 1999 and January−April 2000 | | |
| --- | --- | --- | --- | --- | --- | --- |
|  | | |  |  | | |
| 247 people with 247 personal NO_2_ measurements |  |  |  | 235 people with 236^a^ personal NO_2_ measurements |  |  |
|  |  | *Excluded 5 faulty personal NO_2_ measurements* |  |  |  | *Excluded 12 faulty personal NO_2_ measurements* |
| 242 people with 242 personal NO_2_ measurements |  |  |  | 223 people with 224^a^ personal NO_2_ measurements |  |  |
|  |  | *Excluded 34 people in 3 special exposure groups (34 personal NO_2_ measurements)* |  |  |  | *Excluded 32 people in 3 special exposure groups (33*^a^ *personal NO_2_ measurements)* |
| 208 people with 208 personal NO_2_ measurements |  |  |  | 191 people with 191 personal NO_2_ measurements |  |  |
|  |  | *Excluded 32 people who had missing data on annual work NO_2_ estimates* |  |  |  | *Excluded 14 people who had missing data on annual work NO_2_ estimates* |
|  |  | *Excluded 1 person who had missing data on annual home NO_2_ estimate* |  |  |  | *Excluded 14 people who had missing data on 7-day urban and street NO_2_ levels* |
| 175 people with 175 personal NO_2_ measurements |  |  |  | 163 people with 163 personal NO_2_ measurements |  |  |
|  |  |  |  |  |  |  |
|  |  | 338 people with 338 personal NO_2_ measurements | | |  |  |
|  |  |  | | |  |  |

**Figure S1. Overview of the number of personal NO_2_ measurements included in the regression analyses.**

^a^One person in the ‘Many workplaces’ exposure group had two personal NO_2_ measurements
