# Supplementary material for: Individual Exposure to NO2 in Relation to Spatial and Temporal Exposure Indices in Stockholm, Sweden: The INDEX Study
Source: PLoS One. 2012 Jun 20;7(6):e39536. doi: 10.1371/journal.pone.0039536 (PMC3380030; doi:10.1371/journal.pone.0039536)
Supplement: Table S1 — Multiple regression models: Relationship between individual exposure to NO2, and temporal and spatial indices and time-activity patterns in Stockholm, Sweden. (DOC) [file pone.0039536.s002.doc]

**Table S1. Multiple regression models: Relationship between individual exposure to NO_2_, and temporal and spatial indices and time-activity patterns in Stockholm, Sweden.**

| **Model** | **Coefficient (95% CI)** | ***p*** | **R^2^** |
| --- | --- | --- | --- |
| *Model S1* |  |  | 0.08 |
| 7-day urban NO_2_ (μg/m^3^) | 0.39 (0.25−0.53) | <0.0001 |  |
| Difference between 7-day street and urban NO_2_ (μg/m^3^) | 0.23 (0.15−0.30) | <0.0001 |  |
| Constant | 1.05 (-2.90−5.01) | 0.601 |  |
|  |  |  |  |
| *Model S2* |  |  | 0.03 |
| 7-day rural NO_2_ (μg/m^3^) | 0.35 (-0.03−0.73) | 0.070 |  |
| Difference between 7-day urban and rural NO_2_ (μg/m^3^) | 0.26 (0.11−0.42) | 0.001 |  |
| Constant | 9.05 (5.92−12.18) | <0.0001 |  |
|  |  |  |  |
| *Model S3* |  |  | 0.06 |
| Difference between 7-day street and urban NO_2_ (μg/m^3^) | 0.20 (0.12−0.29) | <0.0001 |  |
| Difference between 7-day urban and rural NO_2_ (μg/m^3^) | 0.32 (0.17−0.47) | <0.0001 |  |
| Constant | 4.14 (0.50−7.78) | 0.026 |  |
|  |  |  |  |
| *Model S4* |  |  | 0.38 |
| Estimated annual NO_2_ at home (μg/m^3^) | 0.31 (0.23−0.39) | <0.0001 |  |
| Estimated annual NO_2_ at work (μg/m^3^) | 0.24 (0.16−0.32) | <0.0001 |  |
| 7-day urban NO_2_ (μg/m^3^) | 0.40 (0.27−0.53) | <0.0001 |  |
| Difference between 7-day street and urban NO_2_ (μg/m^3^) | 0.24 (0.17−0.32) | <0.0001 |  |
| Constant | -7.90 (-11.92− -3.88) | <0.0001 |  |
|  |  |  |  |
| *Model S5* |  |  | 0.31 |
| Estimated annual NO_2_ at home (μg/m^3^) | 0.31 (0.22−0.39) | <0.0001 |  |
| Estimated annual NO_2_ at work (μg/m^3^) | 0.23 (0.14−0.31) | <0.0001 |  |
| 7-day rural NO_2_ (μg/m^3^) | 0.32 (-0.02−0.67) | 0.065 |  |
| Difference between 7-day urban and rural NO_2_ (μg/m^3^) | 0.28 (0.14−0.42) | <0.0001 |  |
| Constant | 0.64 (-2.65−3.94) | 0.703 |  |

**Table S1 (cont.)**

| **Model** | **Coefficient (95% CI)** | ***p*** | **R^2^** |
| --- | --- | --- | --- |
| *Model S6* |  |  | 0.36 |
| Estimated annual NO_2_ at home (μg/m^3^) | 0.31 (0.23−0.39) | <0.0001 |  |
| Estimated annual NO_2_ at work (μg/m^3^) | 0.24 (0.16−0.32) | <0.0001 |  |
| Difference between 7-day street and urban NO_2_ (μg/m^3^) | 0.22 (0.14−0.30) | <0.0001 |  |
| Difference between 7-day urban and rural NO_2_ (μg/m^3^) | 0.34 (0.20−0.47) | <0.0001 |  |
| Constant | -5.02 (-8.77− -1.27) | 0.009 |  |
|  |  |  |  |
| *Model S7* |  |  | 0.38 |
| Estimated annual NO_2_ at home (μg/m^3^) | 0.31 (0.23−0.40) | <0.0001 |  |
| Estimated annual NO_2_ at work (μg/m^3^) | 0.23 (0.15−0.31) | <0.0001 |  |
| 7-day rural NO_2_ (μg/m^3^) | 0.57 (0.24−0.90) | 0.001 |  |
| Difference between 7-day street and urban NO_2_ (μg/m^3^) | 0.25 (0.17−0.33) | <0.0001 |  |
| Difference between 7-day urban and rural NO_2_ (μg/m^3^) | 0.39 (0.25−0.52) | <0.0001 |  |
| Constant | -8.47 (-12.66− -4.27) | <0.0001 |  |
|  |  |  |  |
| *Model S8* |  |  | 0.39 |
| Estimated annual NO_2_ at home (μg/m^3^) | 0.31 (0.23−0.39) | <0.0001 |  |
| Estimated annual NO_2_ at work (μg/m^3^) | 0.24 (0.16−0.32) | <0.0001 |  |
| 7-day urban NO_2_ (μg/m^3^) | 0.42 (0.29−0.55) | <0.0001 |  |
| Difference between 7-day street and urban NO_2_ (μg/m^3^) | 0.23 (0.16−0.31) | <0.0001 |  |
| Time in transport and at a garage (hours/week) | 0.14 (0.03−0.25) | 0.015 |  |
| Constant | -9.57 (-13.79− -5.35) | <0.0001 |  |
|  |  |  |  |
| *Model S9: Sensitivity analysis* |  |  | 0.39 |
| Estimated annual NO_2_ at home (μg/m^3^) | 0.31 (0.23−0.39) | <0.0001 |  |
| Estimated annual NO_2_ at work (μg/m^3^) | 0.24 (0.16−0.32) | <0.0001 |  |
| 7-day urban NO_2_ (μg/m^3^) | 0.42 (0.29−0.55) | <0.0001 |  |
| Difference between 7-day street and urban NO_2_ (μg/m^3^) | 0.24 (0.17−0.32) | <0.0001 |  |
| Time in transport and at a garage (hours/week) | 0.10 (-0.01−0.22) | 0.070 |  |
| Constant | -9.57 (-13.77− -5.37) | <0.0001 |  |

**Table S1 (cont.)**

| **Model** | **Coefficient (95% CI)** | ***p*** | **R^2^** |
| --- | --- | --- | --- |
| *Model S10* |  |  | 0.39 |
| Estimated annual NO_2_ at home (μg/m^3^) | 0.31 (0.23−0.39) | <0.0001 |  |
| Estimated annual NO_2_ at work (μg/m^3^) | 0.23 (0.15−0.31) | <0.0001 |  |
| 7-day rural NO_2_ (μg/m^3^) | 0.59 (0.26−0.92) | 0.001 |  |
| Difference between 7-day street and urban NO_2_ (μg/m^3^) | 0.24 (0.17−0.32) | <0.0001 |  |
| Difference between 7-day urban and rural NO_2_ (μg/m^3^) | 0.41 (0.27−0.54) | <0.0001 |  |
| Time in transport and at a garage (hours/week) | 0.14 (0.03−0.25) | 0.017 |  |
| Constant | -10.21 (-14.62− -5.81) | <0.0001 |  |
|  |  |  |  |
| *Model S11: Sensitivity analysis* |  |  | 0.40 |
| Estimated annual NO_2_ at home (μg/m^3^) | 0.31 (0.23−0.39) | <0.0001 |  |
| Estimated annual NO_2_ at work (μg/m^3^) | 0.23 (0.15−0.31) | <0.0001 |  |
| 7-day rural NO_2_ (μg/m^3^) | 0.59 (0.26−0.92) | <0.0001 |  |
| Difference between 7-day street and urban NO_2_ (μg/m^3^) | 0.26 (0.18−0.33) | <0.0001 |  |
| Difference between 7-day urban and rural NO_2_ (μg/m^3^) | 0.40 (0.27−0.54) | <0.0001 |  |
| Time in transport and at a garage (hours/week) | 0.10 (-0.01−0.22) | 0.081 |  |
| Constant | -10.19 (-14.58− -5.81) | <0.0001 |  |
|  |  |  |  |
| *Model S12* |  |  | 0.40 |
| Estimated annual NO_2_ at home (μg/m^3^) | 0.29 (0.21−0.38) | <0.0001 |  |
| Estimated annual NO_2_ at work (μg/m^3^) | 0.24 (0.16−0.32) | <0.0001 |  |
| 7-day urban NO_2_ (μg/m^3^) | 0.42 (0.29−0.55) | <0.0001 |  |
| Difference between 7-day street and urban NO_2_ (μg/m^3^) | 0.24 (0.16−0.31) | <0.0001 |  |
| Time in transport and at a garage (hours/week) | 0.14 (0.03−0.25) | 0.014 |  |
| Sleeping room window facing a large street | 1.97 (-0.50−4.44) | 0.118 |  |
| Constant | -9.69 (-13.90− -5.48) | <0.0001 |  |

**Table S1 (cont.)**

| **Model** | **Coefficient (95% CI)** | ***p*** | **R^2^** |
| --- | --- | --- | --- |
| *Model S13* |  |  | 0.40 |
| Estimated annual NO_2_ at home (μg/m^3^) | 0.29 (0.21−0.38) | <0.0001 |  |
| Estimated annual NO_2_ at work (μg/m^3^) | 0.24 (0.16−0.32) | <0.0001 |  |
| 7-day rural NO_2_ (μg/m^3^) | 0.56 (0.23−0.89) | 0.001 |  |
| Difference between 7-day street and urban NO_2_ (μg/m^3^) | 0.25 (0.17−0.33) | <0.0001 |  |
| Difference between 7-day urban and rural NO_2_ (μg/m^3^) | 0.41 (0.28−0.55) | <0.0001 |  |
| Time in transport and at a garage (hours/week) | 0.14 (0.03−0.25) | 0.014 |  |
| Sleeping room window facing a large street | 2.16 (-0.32−4.65) | 0.088 |  |
| Constant | -10.33 (-14.73− -5.93) | <0.0001 |  |
