# Supplementary material for: Individual Exposure to NO2 in Relation to Spatial and Temporal Exposure Indices in Stockholm, Sweden: The INDEX Study
Source: PLoS One. 2012 Jun 20;7(6):e39536. doi: 10.1371/journal.pone.0039536 (PMC3380030; doi:10.1371/journal.pone.0039536)
Supplement: Table S2 — Overview of studies of personal exposure to NO2 in random samples of healthy working adult populations. (DOC) [file pone.0039536.s003.doc]

**Table S2. Overview of studies of personal exposure to NO_2_ in random samples of healthy working adult populations**

| **Study area and year** | **Personal exposure level (µg/m^3^)** | **Home outdoor level (µg/m^3^)** | **Sample size** | **Influence of indoor sources** | **Determinants of personal exposure level included in the full model** | **Explanatory power of full model (R^2^)** | **Reference** |
| --- | --- | --- | --- | --- | --- | --- | --- |
| Stockholm, Sweden, 1999-2000, all seasons | 7-day average: 14 (1-2 samples/person) | Annual average: 14 μg/m^3^ | 247 | Excluded a priori gas stove use, | Estimated annual home and work outdoor level, concurrent 7-day street, street minus urban, urban minus rural level, time in traffic, time with gas appliances, time in smoky room, number of days at work | 0.44 | This study |
| Los Angeles, USA, 1987-1988, all seasons | 48-h average: 71 μg/m3 (1 sample/person) | 48-h average: 72 μg/m3 | 682a | Active smoking and ETS exposure excluded a priori, but not gas appliance use | Not investigated | Not investigated | Spengler et al. (1994) |
| SAPALDIA study, in 8 regions in Switzerland, 1993-1994, all seasons | 7-day average: 27 μg/m3  (3 samples/person) | 7-day average 31 μg/m3 | 502 | Gas appliance use, active smoking and ETS not excluded a priori | 7-day home outdoor levels, use of gas stove, active smoking and ventilation included in the model | 0.58 | Monn et al. (1998) |
| 18 cities in 15 countries in Europe, North America and Asia, 1996, winter only | 48-h averages varied from 21 μg/m3) (Geneva, Switzerland) to 97 μg/m3 (Sosnowiec, Poland)  (1 sample/person) | 48-h averages varied from 22 μg/m3) (Geneva, Switzerland) to 98 μg/m3 (Seoul, Korea) | 568 | Gas appliance use, active smoking and ETS not excluded a priori | 48-h home outdoor levels use of gas appliances and ETS | 0.45 | Levy et al. (1998) |

**Table S2 (cont.)**

| **Study area and year** | **Personal exposure level (µg/m^3^)** | **Home outdoor level (µg/m^3^)** | **Sample size** | **Influence of indoor sources** | **Determinants of personal exposure level included in the full model** | **Explanatory power of full model (R^2^)** | **Reference** |
| --- | --- | --- | --- | --- | --- | --- | --- |
| EXPOLIS study, 1996-1997, all seasons | 48-h average: 30 μg/m^3^ (Basel, Switzerland), 25 μg/m^3^ (Helsinki, Finland) , 43 μg/m^3^ (Prague, Czech republic)  (1 sample/person) | 48-h averages: 24 μg/m^3^ (Helsinki), 36 μg/m^3^ (Basel), 61 μg/m^3^ (Prague) | 201 (Basel),  50 (Helsinki), 35 (Prague)^b^ | Gas appliance use, active smoking and ETS not excluded a priori | 48-h home outdoor levels, work location, use of gas appliances, keeping windows open | 0.48 | Kousa et al. (2001) |
| Oxford, UK, 1998-2000, all seasons | 48-h average: 29 μg/m^3^  (1 sample/person) | 48-h average: 27 μg/m^3^ | 50 | Gas appliance use, active smoking and ETS not excluded a priori | Not investigated | Not investigated | Lai et al. (2004) |
| Detroit, USA, 2004-2007, all seasons | 24-h average: 26 μg/m^3^ (summer), 36 μg/m^3^ (winter)  (On average 8 samples/person) | Not reported | 136^c^ | Active smoking excluded a priori, but not ETS and gas appliance use | Urban background home air exchange, dryer pilot light, air conditioning, use of gas stove, forced air gas furnace, open windows | 0.12 | Williams et al. (2011) |

^a^Randomly selected population: 13% younger than 19 years, 68% between 20-60 years, 19% over 60 years. About 33% did not have a regular out-of-home activity, 13% were students, 54% were employed.

^b^Volunteers (adults), not randomly selected health working population.

^c^Randomly selected population: About 66% were not employed (either retired or stay-at-home residents). Aged 18-79 years, mean 42 years.
