# Supplementary material for: Individual Exposure to NO2 in Relation to Spatial and Temporal Exposure Indices in Stockholm, Sweden: The INDEX Study
Source: PLoS One. 2012 Jun 20;7(6):e39536. doi: 10.1371/journal.pone.0039536 (PMC3380030; doi:10.1371/journal.pone.0039536)
Supplement: Text S1 — Tests applied to determine significance of differences in personal NO2 levels. (DOC) [file pone.0039536.s004.doc]

***Text S1***

According to the Shapiro-Wilk test, the 7-day personal NO_2_ levels, 7-day outdoor street, urban and rural NO_2_ levels, and the annual outdoor NO_2_ estimates at home and work did not have normal Gaussian distributions. Hence, non-parametric tests were applied. Wilcoxon’s rank-sum tests (also known as the Mann-Whitney two-sample statistic) were applied to test whether personal NO_2_ levels differed significantly between the study participants who lived in the city compared to those who lived outside the city. Wilcoxon’s rank-sum tests were also done to test whether the personal NO_2_ levels differed significantly between the study participants who work in the city compared to those who worked outside the city, after stratifying by home location. Kruskal-Wallis tests were conducted to test whether personal NO_2_ levels differed significantly across the transport groups, after stratifying by home and work location (Groups 1 to 14, Figure 1).
