# Supplementary material for: Individual Exposure to NO2 in Relation to Spatial and Temporal Exposure Indices in Stockholm, Sweden: The INDEX Study
Source: PLoS One. 2012 Jun 20;7(6):e39536. doi: 10.1371/journal.pone.0039536 (PMC3380030; doi:10.1371/journal.pone.0039536)
Supplement: Text S2 — Extreme observations for time-activity variables. (DOC) [file pone.0039536.s005.doc]

***Text S2***

One extreme observation of time spent in places with gas appliances during the 7-day measurement period (in a room with a refrigerator powered by a liquid gas flame) (24 h) and one extreme of time spent in traffic during the 7-day measurement period (32.5 h) were identified. We judged these values to be valid and chose not to exclude them in the statistical analyses. We did a sensitivity analysis and also excluded these two observations from the regression analyses.
